# Navigating Oral Hygiene Challenges in Spastic Cerebral Palsy Patients: A Narrative Review for Management Strategies for Optimal Dental Care

**DOI:** 10.7759/cureus.50246

**Published:** 2023-12-09

**Authors:** Sucharitha Palanisamy, Priyanka Cholan, Lakshmi Ramachandran, Anupama Tadepalli, Harinath Parthasarsthy, Santo G Umesh

**Affiliations:** 1 Periodontics & Oral Implantology, SRM Dental College & Hospital, Chennai, IND

**Keywords:** toothbrush modifications, oral health management, intellectual disability, spasticity, cerebral palsy

## Abstract

In the realm of well-being, the essence of maintaining optimal oral health is gaining more recognition. This quantifying quotient is being compromised in cerebral palsy (CP) patients due to multitude variations. Spastic CP predominantly impacts bodily motions, muscle synchronization, command, muscle tone, reflexes, stance, equilibrium, and can additionally influence both delicate and large-scale motor abilities. For individuals with spastic CP, the rigidity extends its influence over both their upper and lower limbs. When this stiffness takes hold in the upper limb, it poses significant challenges in executing everyday activities, causing issues with precise grasping and coordination of muscle movements. Consequently, using a toothbrush effectively becomes a formidable task resulting in widespread caries and periodontal diseases in spastic CP patients. The central focus of this review is to explore the oral health challenges of spastic cerebral palsy patients and mapping out a path towards the most efficient time-tested and innovative dental management approaches for preserving oral health in these patients.

## Introduction and background

Cerebral palsy (CP) encompasses a cluster of neurological conditions that manifest during infancy or early childhood, leading to enduring impacts on the coordination of muscles and body movement [1]. It emerges due to damage or irregularities within the evolving brain, impeding its capacity to regulate motion, posture, and equilibrium. Within the International Classification of Diseases (ICD), numerous systemic illnesses are identified by specific codes. CP stands among these conditions, focusing on cognitive impairments, and is notably referenced in the ICD-10 as G80 [2]. Optimal oral health is a cornerstone of general wellness and unfortunately, individuals with spastic CP frequently encounter considerable hurdles in upholding adequate oral hygiene. This is chiefly attributed to the upper limb spasticity that impedes their capacity to engage in effective oral care practices. Among individuals with spastic CP, a spectrum of issues arise, encompassing alterations to the orofacial region's physical structure, neuromuscular limitations, feeding difficulties, struggles in upholding oral cleanliness, and obstacles in obtaining dental care. The central catalyst of suboptimal oral hygiene in spastic CP patients predominantly emerges from upper limb spasticity, which impedes their ability to grasp a toothbrush effectively and carry out efficient oral care routines [3].

The root cause of spasticity lies in the damage inflicted upon the motor cortex and the pyramidal tracts within the brain. These neural pathways act as the bridge connecting the motor cortex and the CNS, enabling the transmission of signals that orchestrate movement. When injuries affect this area, it impedes the capacity of individuals with spastic CP to effectively reach out and seize objects, including toothbrushes. Spasticity in cerebral palsy patients manifests as abrupt, erratic movements, muscular tension, and rigidity in the joints. In the upper limbs, it presents as the bending of the elbow, the curving of the wrist, and difficulties coordinating grip strength and the application of force in the fingers [4]. In the realm of CP, the motor cortex takes on a pivotal role in regulating voluntary bodily actions, and damage to this brain region results in loss of precision and fluidity in voluntary movement, rendering it "spastic." Achieving a precise grip on the toothbrush necessitates a delicate balance of force applied right angle to the interface surfaces (known as grip force) and the counteracting tangential load forces that oppose gravity's effects. In individuals with paretic hands affected by spastic CP, this grip-lift motion displays characteristics such as a lack of synchronization between the initiation of grasp and pressure forces, diverse successive increases in traction and tangential force instances (known as force derivatives), and additional grasp force particularly when tangential load begins to rise and consequently compromises the ability to efficiently grasp objects [5].

Individuals with CP frequently experience challenges in managing the intricate mechanics of their oral motor function. In simpler terms, they encounter difficulties in taming the muscles in their mouth and throat. This struggle often manifests as hurdles in the realm of nourishment, making actions like sucking and chewing a bit of a quest. CP patients face a heightened vulnerability to dysphagia as a result of their struggles with muscle and motor function control [6]. These difficulties can manifest through a range of symptoms, such as difficulty in initiating swallowing, often accompanied by painful sensations, episodes of regurgitation, unpleasant bouts of heartburn, sensations of stomach acid creeping up into the throat, unexplained and atypical weight loss, hoarse quality to their voice, sensation of food getting stuck in the chest or throat, occasional bouts of gagging and coughing during swallowing attempts, an increase in drooling, delayed or even absent swallowing reflex, persistent sore throat. This added complexity can make it tough for them to ensure proper oral care, ultimately resulting in an increased buildup and retention of dental biofilm, which poses a risk to their dental health [7,8].

Managing the dental health of people with spastic CP poses a significant challenge for both caregivers and dental professionals, and the nature of these challenges varies depending on the severity of cognitive and physical impairments [9]. The involvement of dental hygienists and the development of home-based dental care routines play crucial roles in restoring dental hygiene in such individuals. This article sheds light on various innovative dental management strategies dedicated to enhancing individuals' oral health with CP.

## Review

Oral manifestations in cerebral palsy

**Figure 1:**
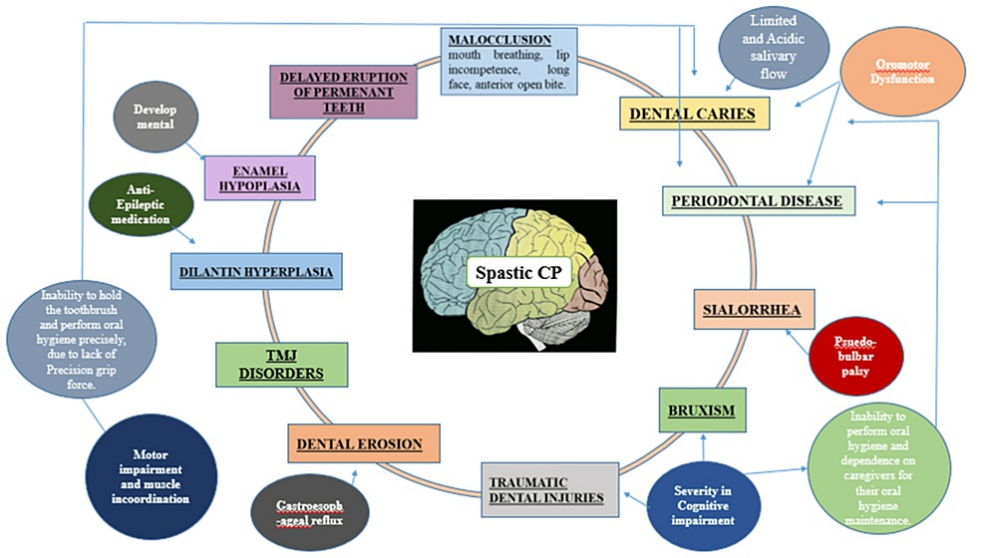
Predisposing factors and oral manifestations in spastic cerebral palsy (CP) patients. Image credit: Self-made figure by the author.

Sehrawat et al. (2014) unveiled the intricate neuromuscular obstacles manifested in CP, which exerted a substantial influence on oral health through various channels [10]. These factors encompass changes in the orofacial region's anatomy, challenges associated with feeding, challenges in maintaining effective oral hygiene, and obstacles in accessing dental health care for spastic CP patients [11,12]. The factors that set the stage for these challenges included motor impairment coupled with muscle coordination issues, oromotor dysfunction, pseudobulbar palsy, gastroesophageal reflux, and nutritional deficiencies, particularly in vitamin D are shown in Figure 1.

Evidence-based practices in dental management of spastic cerebral palsy patients

This encompasses preventive care, surveillance, and recovery. The dental care approaches can be categorized into two groups: conservative methods and innovative evidence-based strategies, depending on their chronological sequence.

Conventional Management Regimes 

Oral hygiene patterns: Wyne et al. (2017) emphasized the paramount importance of upholding oral hygiene maintenance strategies for individuals with spastic CP. Both CP patients and their caregivers must commit to daily brushing and flossing of their teeth. This regular brushing routine, done twice a day, is essential for preserving a healthy oral cavity. By diligently removing plaque daily, the likelihood of developing new cavities should be significantly reduced. Enhancing oral hygiene habits at home can lead to a decrease in tooth erosion, plaque buildup, and malocclusions, as well as a reduction in measures like decayed, missing due to caries, and filled teeth (DMFT) in this population. Caregivers and children with CP may need thorough oral hygiene guidance, coupled with periodic reinforcement. If a child faces difficulties in performing these actions, it becomes the responsibility of parents or caregivers to oversee the practice until proficiency is achieved [13].

Kachwinya et al. (2022) highlighted the obstacles that impede CP patients from maintaining proper oral hygiene. They emphasized the importance of motivating patients to embrace positive brushing and flossing habits, while also increasing awareness among parents and caregivers about the significance of oral hygiene [14]. Moreover, Akhter et al. (2019) focused on the association between oral health and general well-being of life in people with spastic CP. They accentuated the significance of adapting oral hygiene habits and creating awareness among cerebral palsy patients about proper oral health care practices [15].

Fluorides: The Australian Dental Association (ADA) has undertaken a thorough exploration of various fluoride-based preventive measures, such as mouthwashes, sealants, and dentifrices. These options are seen as a safe and effective clinical strategy for slowing down or even stopping the progression of dental decay. Depending on the ADA's clinical recommendations and the practitioner's skill, these treatments can be administered either at home or in a clinical setting. Incorporating fluoride into the oral care regimen of children with spastic CP can function as a robust defense against the demineralization process, which is often initiated by the presence of food remnants and harmful bacteria [16].

Diet modification: Ahmad et al. (2019) conducted an assessment of the dental health condition and nutritional well-being of spastic CP patients. The feeding challenges faced by these children stem from underdeveloped neurological and muscular functions, resulting in poor eating efficiency due to compromised oral skills. Their difficulty in executing regular swallowing motions involving the tongue, lips, and cheeks impedes proper food consumption, leaving remnants in the mouth [17].

To counteract the cariogenic impact of certain foods, caretakers are recommended to keep an eye on their children from frequent snacking and the consumption of a non-solid, carbohydrate diet. In the pursuit of enhancing dietary effectiveness in this population, future evaluations should explore the introduction of non-solid supplements to supplement standard nutrition [18]. This may necessitate the use of various sugar substitutes to achieve a balance between stability, taste onset, and sweetness intensity. These changes hold the potential for significant public health benefits by reducing the incidence of dental caries among spastic CP patients. Furthermore, Pani et al. (2020) emphasized the enhancement of dental health status through modifications in oral hygiene practices and dietary adjustments like changes in food texture and beverage thickness so that they can swallow safely [19].

Restorative treatment: If the advancement of dental caries is not effectively controlled, it can lead to the formation of lesions. These lesions are particularly prone to develop in plaque retentive aspects found on the chewing surfaces of permanent posterior teeth, where food particles can become trapped and create a conducive environment for bacterial biofilm growth. Consequently, it is essential to employ secondary preventive measures to slow down the advancement of these caries [20].

The utilization of fissure sealants, temporary glass ionomer cement fillings or varnish applications, the removal of damaged enamel, dentine conditioning, or varnish application are a few instances of these management techniques. The primary objective of lesion management is to prepare the oral environment for caries prevention, reduce the risk of bacterial infections, and prevent food particles from getting trapped in open cavities. The utilization of mouth props, ensuring the dental chair is correctly positioned, and employing rubber dams for isolation during dental procedures are of paramount significance. If patients experience discomfort with rubber dams, it is advisable to introduce them gradually until they become at ease with the technique [21].

Katz et al. (2012) introduced an integrated outpatient dental treatment approach for CP patients. This holistic method emphasized a well-rounded strategy for dental care in these patients. Remarkably, it yielded improved motor development in the child, attributable to the collaborative efforts of the dental hygienist, physiotherapist, and verbal communication rehabilitation, alongside the active participation of caregivers, who continued to provide stimulation at home. This comprehensive approach made a substantial positive impact on overall oral health status [22].

Toothbrush modification: Some individuals diagnosed with CP can independently manage their teeth cleaning routine, necessitating only minor adjustments to a standard toothbrush. For these individuals, incorporating a larger grip onto the toothbrush allows them to handle and maneuver the brush autonomously. 

Achieving this adaptation can be accomplished through several methods like acquiring a toothbrush with a wider handle, as certain companies produce toothbrushes with larger handles designed to offer an improved grip, creating an incision in a tennis ball, inserting the base of the toothbrush handle into the cut tennis ball, placing the toothbrush base into a bicycle handle grip or using rubber or foam tubing to enhance the grasp, wrapping a small cloth around the bottom of the brush to increase grip and control [23].

Adjustments to the toothbrush shape to achieve a more favorable angle for accessing their teeth. This can be accomplished through the following methods which includes obtaining a toothbrush designed with a curved shape that facilitates more effective brushing angles. Some companies offer such bendable brushes, readily available at local convenience stores, running the toothbrush handle (while avoiding contact with the bristles) under very hot water, allows for the gentle reshaping of the plastic to meet the specific requirements of the individual.

Certain CP individuals may require an extended toothbrush to effectively reach their mouth for independent brushing. This can be achieved by extending the toothbrush using the following materials: Attaching multiple popsicle sticks to the base of the brush with tape and adding a type of tubing to the bottom for increased length.

Yitzhak et al. (2012) investigated the two distinct toothbrush designs against each other which includes one with three heads and the other with a single head. The results showed that the 3-headed toothbrush surpassed the single-head toothbrush, resulting in more significant reductions in both plaque index (PI) and modified gingival index (MGI) [24]. Ferraz et al. (2014) evaluated the effectiveness of various brushing techniques, including manual brushing, continuous electric toothbrush use, and alternating between electric and manual toothbrushes. Remarkably, all three brushing methods resulted in a significant reduction in biofilm accumulation [25]. 

Rehabilitation for spasticity facilitating the optimal grip force: Gunel et al. (2009) introduced a comprehensive, multidisciplinary approach to address disabilities or limitations stemming from physical, mental, sensory-perceptual, or cognitive disorders caused by factors before birth, during birth, or after birth. Within this approach, dental therapy for individuals with physical and mental challenges should be integrated as a standard component of the rehabilitation process.

Gunel et al. (2011) emphasized the necessity of a multidisciplinary team (MDT) approach in pediatric rehabilitation. This approach is crucial for fostering the independence of children with impairments, addressing both their functional and psychological needs. The primary objective of employing a multidisciplinary approach to physiotherapy was to alleviate muscle stiffness, regulate reflex responses, and facilitate proper gripping abilities for holding objects securely. Furthermore, sensory integration techniques can contribute to enhancing fine-touch sensitivity and precision in actions [26].

Interventions aimed at improving hand function encompass a range of strategies, including constraint-induced movement therapy (CIMT), occupational therapy, hand-arm bilateral intensive therapy (HABIT), splinting, Botox injections, and surgical procedures.

Reinforcements like teleconferencing: Katz et al. (2012) underscored the significant role of teleconferencing in instructing individuals on their oral hygiene practices. Teleconferencing predominantly focuses on the duration, frequency, and self-motivation of oral hygiene practices. The duration and frequency of teleconferencing depend on the patient’s performance and motivation towards the maintenance of oral hygiene. Guiding teleconferencing not only reaffirms the methods they employ but also trains them to consistently carry out their oral care routines [22].

Parental and caregivers perspective on spastic cerebral palsy patients: Puthiyapurayil et al. (2022) examined how parents and caregivers perceive the oral health conditions of individuals with CP. Data was collected through a questionnaire known as P-CPQ. The findings indicated that parents of children falling into the high-severity DMFT category reported more elevated scores in the oral health-related quality of life (OHRQoL) domains [27]. Selva et al. (2022) focused on semi-structured forms to evaluate the barriers to access to oral hygiene care. The more severe the CP, the greater the difficulty of accessibility, and the lower the income, the greater the motor impairment. Despite the availability of access to dental services, low-income families have more severe CP patients, contributing to the daily difficulties already faced in oral health care [28]. Sruthi et al. (2021) conducted an oral examination to evaluate and draw comparisons between the oral health conditions and the way parents perceive their children's OHRQoL in two groups: children with CP and those without it. The results stated that children with CP had significantly higher rates of dental trauma, gingivitis, dental cavities, and erosion [29].

Hamid et al. (2017) focused on parental knowledge, attitudes, and practices concerning the dental health status in CP individuals. These investigations were conducted via face-to-face interviews employing questionnaires. The results unveiled that caries prevalence is higher in primary teeth than in permanent teeth [30]. Abanto et al. (2012) analyzed the effect of disabilities and oral health using a comprehensive OHRQoL. This tool used P-CPQ and FIS. The findings highlighted that dental caries, communication skills, and family income were strongly linked to a detrimental effect on oral health [31].

American Academy of Pediatric Dentistry provided the definition of special health care needs in 2016 [32]. The guidelines are mentioned in Table 1.

**Table 1:** American Academy of Pediatric Dentistry (AAPD) guidelines for special care needs

S.NO	AAPD GUIDELINES
1	Establishing a dental home at an early age
2	Obtaining thorough medical, dental, and social patient histories
3	Creating an environment conducive for the child to receive care
4	Providing comprehensive oral health education and anticipatory guidance to the child and caregiver
5	Providing preventive and therapeutic services including behavior guidance and a multidisciplinary approach when needed

Dental clinic considerations for spastic cerebral palsy patients

When dealing with dental checkups or treatments for children diagnosed with CP, several essential factors should be taken into account. Often, practical challenges arise, such as dealing with their anxiety, fear of strangers, limited cognitive abilities, difficulty focusing, and communication obstacles. If the child can adapt to the environment in the dental clinic comfortably, they can be approached much like any other patient. However, those with limited physical control may require additional assistance [33].

Proper adjustment of the dental chair is crucial, with many of these patients benefiting from a reclined position that provides a sense of security. Particularly for spastic patients with significant head and neck involvement, extra control and support may be needed, even allowing them to sit on a parent's or assistant's lap, leaning against their right shoulder. If the patient is using a wheelchair, it's important to consider providing treatment in the wheelchair itself. The initial visit may not involve significant dental work but should primarily focus on building mutual trust and conducting a preliminary assessment. 

Scheduling early in the day allows for sufficient time to establish a comfortable interaction during these encounters. Occasionally, obtaining dental X-rays may require assistance from the parent, a dental assistant, or the use of immobilization devices. A supportive and calm approach from the dentist can greatly enhance the child's cooperation. Maintaining the head position of CP patients in the midline can be achieved with the help of Velcro straps, while mouth props can assist in keeping the mouth open during treatment. 

Dentists should aim to be gentle and compassionate, avoiding sudden movements that could trigger muscle stiffness or spasms. To prevent injury, it's advisable to use a finger guard and a steel mirror, exercising extreme caution when employing sharp instruments. For immobilization, canvas straps with Velcro fasteners can be employed to secure the arms and legs, encircling the limbs and the footrest of the dental chair. Placing towels over the arms or legs can protect the patient's skin. Alternatively, wrapping a sheet around the patient can also be an effective immobilization method. Taking a preventive approach should involve a collaborative effort among dentists, hygienists, assistants, patients, families, and other individuals who have an impact on the patient's life [34].

Evidence-based newer oral health management strategies for spastic cerebral palsy patients

The emerging strategies in oral management will shift their emphasis towards specific aspects rather than adopting a broad, all-encompassing approach. This approach will prioritize the removal of etiopathological factors and enhance the effectiveness of oral hygiene practices. The evidence based newer oral health management strategies for spastic cerebral palsy patients are mentioned in Table 2.

**Table 2:** The compilation of literature evidence regarding innovative approaches to maintaining oral hygiene in individuals with cerebral palsy (CP) and intellectual disabilities. TQHPI - Turesky Quigley Hein plaque index, PI - plaque index, MGI - modified gingival index, SRP - scaling and root planing, GI - gingival index, VVIM - visual-verbal integration model, RCT - randomised controlled trials, NRS - non-randomised studies, ID - intellectual disability

AUTHOR and YEAR	TITLE	ORAL HYGIENE TOOL/ MODIFICATIONS	INFERENCE
Nour Asaad et al. 2022 [35]	Effectiveness of Apple Cider Vinegar and Mechanical Removal on Dental Plaque in CP patients.	Apple cider vinegar + SRP	In this research, the assessment focused on plaque buildup and gingival health using the TQHPI and MGI. Notable reduction in both indices in the apple cider vinegar group from the beginning of the study to the 6-month mark (p<0.05).
Burhanuddin Daeng Pasiga et al. 2020 [36]	Utilization of Special Grip Toothbrushes for CP patients.	Toothbrushes with specific handles	In this research, the focus was on assessing the dental hygiene status and the presence of halitosis in CP patients. A toothbrush specially crafted with a unique handle design made out of clay was employed. The distinct design of this toothbrush made it easier for individuals to effectively uphold their oral hygiene practices, leading to a reduction in bad breath.
Lakshmi Krishnan et al. 2019 [37]	Effectiveness of Two Different Modifications of Manual Toothbrush on Plaque Control in CP patients.	Toothbrushes with modified handle and head	In this study, adaptations were made to the standard toothbrush, encompassing changes to both its handle and head. The alterations made to the toothbrush involved using a 5mm ethaflex sheet to achieve the necessary thickness (corresponding to the child's hand grip circumference). The second adjustment included heating the brush with a hot air gun for 15 seconds, followed by angling it at 35˚-40˚ using a protractor at the shank. The alterations implemented on the toothbrush's handle resulted in a statistically notable variance in the PI. The manual brushing adjustments proved equally as efficient in removing plaque when juxtaposed with conventional brushing methods.
Husna Afifah et al. 2019 [38]	Effectiveness of wall-mounted automatic toothbrush against oral hygiene on CP patients.	Wall-mounted automatic toothbrush	In this study, modifications were introduced to the conventional toothbrush, encompassing alterations to both the brush's handle and head. Automated wall-mounted toothbrush systems feature a toothpaste dispenser for applying toothpaste to the brush and a water sprayer for rinsing after the completion of the tooth-brushing process. These adjustments applied to the toothbrush's handle yielded a statistically significant difference in the PI. The research demonstrated that these manual brushing modifications were just as proficient in removing plaque as the traditional brushing techniques.
Ni Zhou et al. 2019 [39]	Effectiveness of a visual-verbal integration model in training parents and their preschool children with intellectual and developmental disabilities to dispense a pea-sized amount of fluoridated toothpaste.	Visual-verbal integration model	This research centered on assessing the impact of visual-verbal integration models in coaching parents and their intellectually challenged children. The training utilizing visual-verbal integration models notably enhanced parents' proficiency in dispensing toothpaste. Moreover, children displaying greater adaptive skills experienced advantages from the VVIM training.
Catherine Waldron et al. 2019 [40]	Oral hygiene interventions for intellectual disabilities patients.	Various oral hygiene interventions (Cochrane study)	This study scrutinized the impacts, both positive and negative, of oral hygiene interventions, particularly the mechanical elimination of plaque, for ID patients. The research encompassed 19 RCTs and 15 NRSs comprising 1795 adults and children with ID along with 354 caregivers. Multiple approaches have been reviewed, encompassing distinctive manual and powered toothbrushes, oral hygiene education, monitored toothbrushing during programmed dental checkups, discussion using clinical photographs of plaque, different toothbrushing frequencies, plaque-disclosing agents, and personalized care strategies. The study found moderate-certainty evidence supporting only one conclusion: electric and manual toothbrushes are likely similarly effective in decreasing gum inflammation in people with ID over the mid-term. However, further comprehensive research is necessary to fully appraise interventions that display potential for enhancing oral hygiene among individuals with ID. It is crucial to confirm which interventions might be ineffective. Meanwhile, any amendments to current practices based on this review should be done with caution. Decisions about oral hygiene care should be based on professional expertise as well as the particular requirements and preferences of ID patients and their caregivers.
Trupthi Rai et al. 2018 [41]	Evaluation of the effectiveness of a custom-made toothbrush in maintaining oral hygiene in CP patients.	Customized toothbrushes	In this study, the effectiveness of personalized toothbrushes was compared to standard oral hygiene practices in preserving both dental and gum health in individuals with cerebral palsy. These personalized toothbrushes included features such as customized grip designs, elongated handles, electric toothbrushes with wider grip holders, and toothbrush heads designed with double or triple heads and longer shanks. Remarkably, the group using tailored toothbrushes displayed a notable reduction in percentages when examining the initial and final measurements for both the Plaque Index (PI) and the Modified Gingival Index (MGI).
Betty Saptiwi et al. 2018 [42]	The Impact of Irene's Donuts' Innovative School Program on Children with Special Needs Oral Health Care and Hygiene Index.	Oral hygiene maintenance program	In this study, the impact of ID’s Oral Health School Innovative Program on diminishing the risk of cavities in children with special needs was centered. This pioneering program emphasizes the significance of oral hygiene for both parents/caregivers and young patients. Clinical measures were documented before and after the behavioral program. A noticeable contrast in the Oral Hygiene Index Simplified (OHI-S) was observed before and after the behavioral program, demonstrating substantial enhancements particularly in parental/caregiver awareness regarding the importance of oral hygiene.
S M Kalf-Scholte et al. 2018 [43]	Plaque removal with triple-headed vs single-headed manual toothbrushes-a systematic review.	Triple-head vs conventional manual toothbrushes	In this research the efficiency of triple-headed versus single-headed manual toothbrushes in eliminating plaque is evaluated. The triple-headed toothbrush has three flexible and independent heads that cover the palatal (45-degree angle), buccal (45-degree angle), and occlusal surfaces attached to a single shank, ensuring comprehensive cleansing within that specific dental arch. Opting for a triple-head manual toothbrush, over conventional toothbrush, could be advantageous for plaque removal, especially when a caregiver is responsible for brushing a care-dependent individual.
Ana García-Carrillo et al. 2016 [44]	Use of Manual toothbrush vs sonic powered toothbrush in patients with intellectual disability: a cluster-RCT.	Sonic-powered or manual toothbrush	The efficacy of a sonic-powered or manual toothbrush in ID patients was evaluated in the context of PI and GI indices, alongside potential risks. The sonic-powered toothbrush validated was approximately as effective and safe as the manual toothbrush. In patients with mild to moderate ID, using powered or manual toothbrushes in conjunction with fluoride toothpaste might be beneficial to mitigate plaque and gingivitis.

## Conclusions

The past and current methods for preserving oral hygiene in cerebral palsy patients have displayed a modest improvement in oral health, yet these approaches have been quite generic. Significant enhancements in oral health can be achieved when the treatment strategies become more tailor-made rather than one-size-fits-all. In the future, it's essential to direct our attention towards tackling the individual factors in play, such as variations in grip force dynamics, the extent of spasticity, levels of motivation concerning oral health, and specific oromotor hurdles like dysphagia and food retention. These holistic oral health rehabilitation programs should prioritize the preservation of the doctor-caregiver-child relationship, as this can help restore family dynamics and subsequently elevate their overall health. 
